# Supplementary material for: Unified AI framework to uncover deep interrelationships between gene expression and Alzheimer’s disease neuropathologies
Source: Nat Commun. 2021 Sep 10;12:5369. doi: 10.1038/s41467-021-25680-7 (PMC8433314; doi:10.1038/s41467-021-25680-7)
Supplement: Supplementary file 1 — Supplementary Information [file 41467_2021_25680_MOESM1_ESM.pdf]

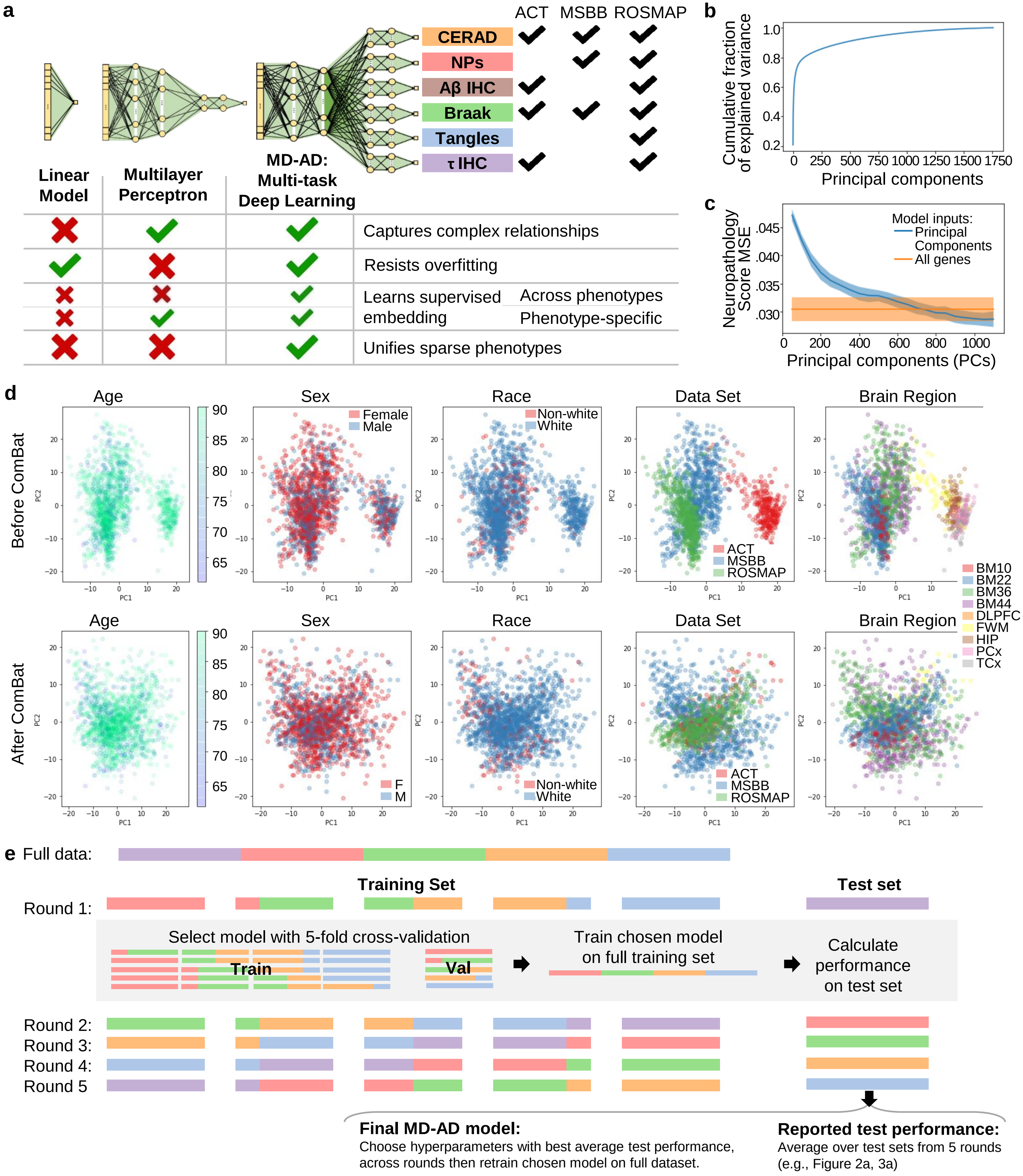

Supplementary Figure 1. (a) Overview of MD-AD and its advantages over traditional approaches. **(b)** Cumulative variance explained for principal components (computed from the full dataset after all pre-processing). (**c**) Average test MSE for predicting average neuropathology score from linear model trained on PC-transformed inputs vs all genes with standard error bands (n=5 test runs). (**d**) First two principal components of gene expression data before and after ComBat batch effect correction. Brain regions shown: Brodmann areas 10, 22, 36, 44 (BM10, BM22, BM36, BM44), dorsolateral prefrontal cortex (DLPFC), hippocampus (HIP), frontal white matter (FWM), parietal cortex (PCx), temporal cortex (TCx). **(e)** Overview of our cross-validation (CV) and testing scheme. We generate five separate training and test splits, and then in each round, we perform cross-validation to choose hyperparameters, retrain the model on the full training set, and then report test performance.

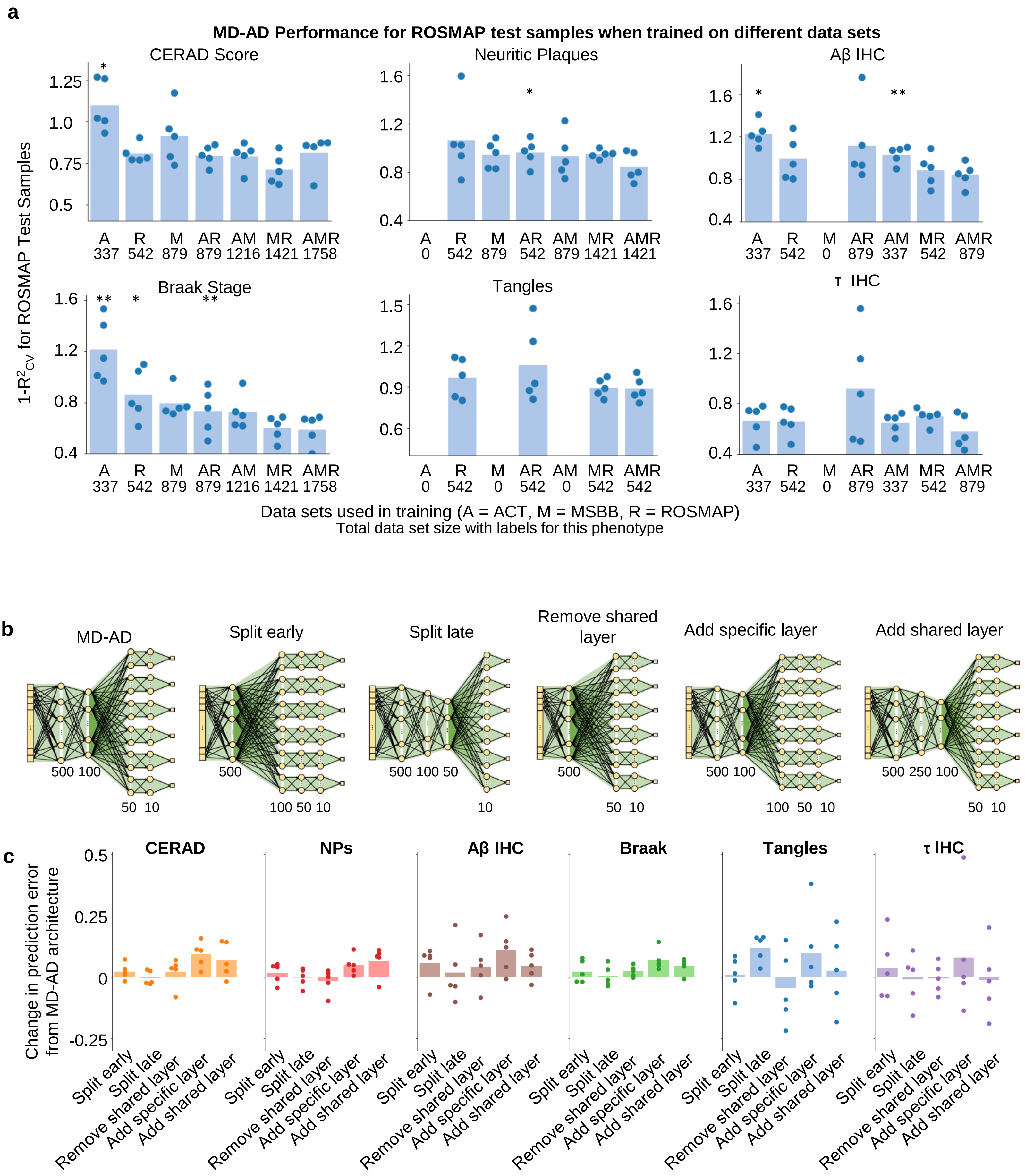

Supplementary Figure 2. (a) We evaluate test set performance for ROSMAP using the same training and test splits, but training restricted to different subsets of available data sets, averaged over test folds (n=5 test runs per data set). **(b)** We experimented with several architectures for the MD-AD model. They are depicted with associated dense layer sizes. (**c**) We plot the difference in test set prediction error between five alternative architectures evaluated and the final selected MD-AD architecture, averaged over five test folds. Positive values indicate higher error relative to the MD-AD architecture (n=5 test runs per architecture).

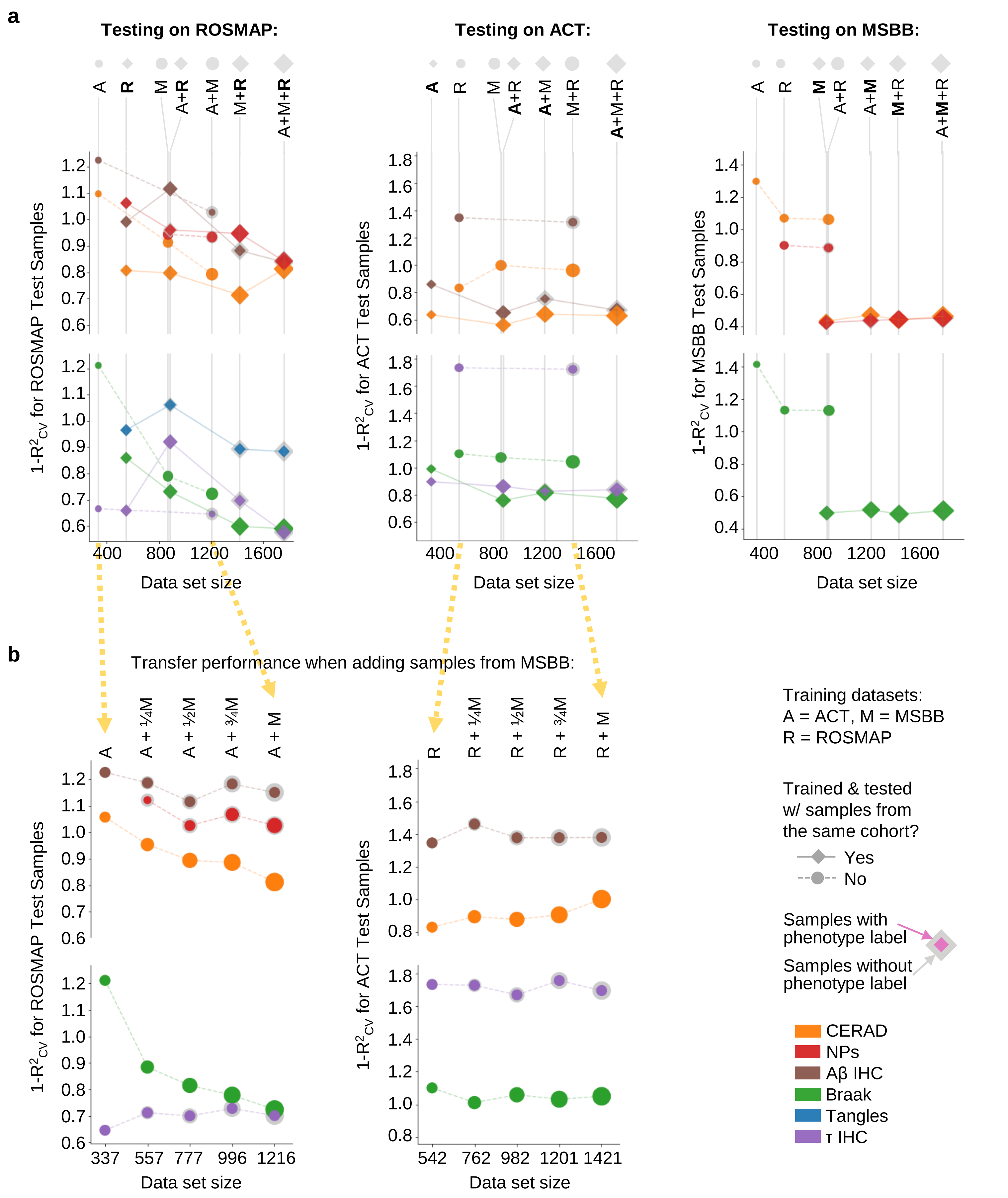

Supplementary Figure 3. **(a)** Test prediction performance using 5-fold cross-validation when training on different subsets of available datasets. We display performance for each dataset's test samples separately. Circle markers show performance when transferring a trained model to a new dataset; diamond markers show changes in performance when augmenting the training set with samples from other datasets. **(b)** Same analysis as part (a), but highlighting how transfer performance changes when adding additional MSBB samples during training.

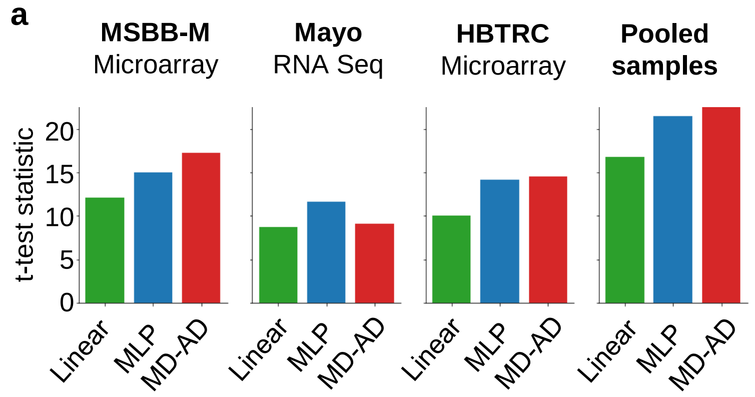

b

		<75		75-85		>85	
		C	AD	C	AD	C	AD
<75	C (179)		4e-28	0.005	1e-35	1e-08	5e-61
	AD (95)	4e-28		3e-18	0.011	2e-23	
75-85	C (134)	0.005	3e-18		5e-20		2e-34
	AD (243)	1e-35	0.011	5e-20		3e-25	0.03
>85	C (337)	1e-08	2e-23		3e-25		8e-50
	AD (554)	5e-61		2e-34	0.03	8e-50	

t-test *p*-value: <.05 <.01 <.001 Not significant

c

		2 months			4 months			8 months			18 months		
	Group (N)	WT	HET	HO	WT	HET	HO	WT	HET	HO	WT	HET	HO
2 m	WT (22)				.021	.009	2e-4	.002	2e-4	1e-6	2e-5	2e-6	1e-8
	HET (8)				.028	.029	7e-4	.005	9e-5	4e-5	3e-4	4e-5	9e-8
	HO (8)									.015		.016	.002
4 m	WT (20)	.021	.028							.007		.008	6e-4
	HET (8)	.009	.029										
	HO (8)	2e-4	7e-4										.015
8 m	WT (20)	.002	.005							.02		.025	.002
	HET (8)	2e-4	9e-5										4e-4
	HO (8)	1e-6	4e-5	.015	.007			.02					
18 m	WT (14)	2e-5	3e-4										.018
	HET (8)	2e-6	4e-5	.016	.008			.025					
	HO (6)	1e-8	9e-8	.002	6e-4		.015	.002	4e-4		.018		

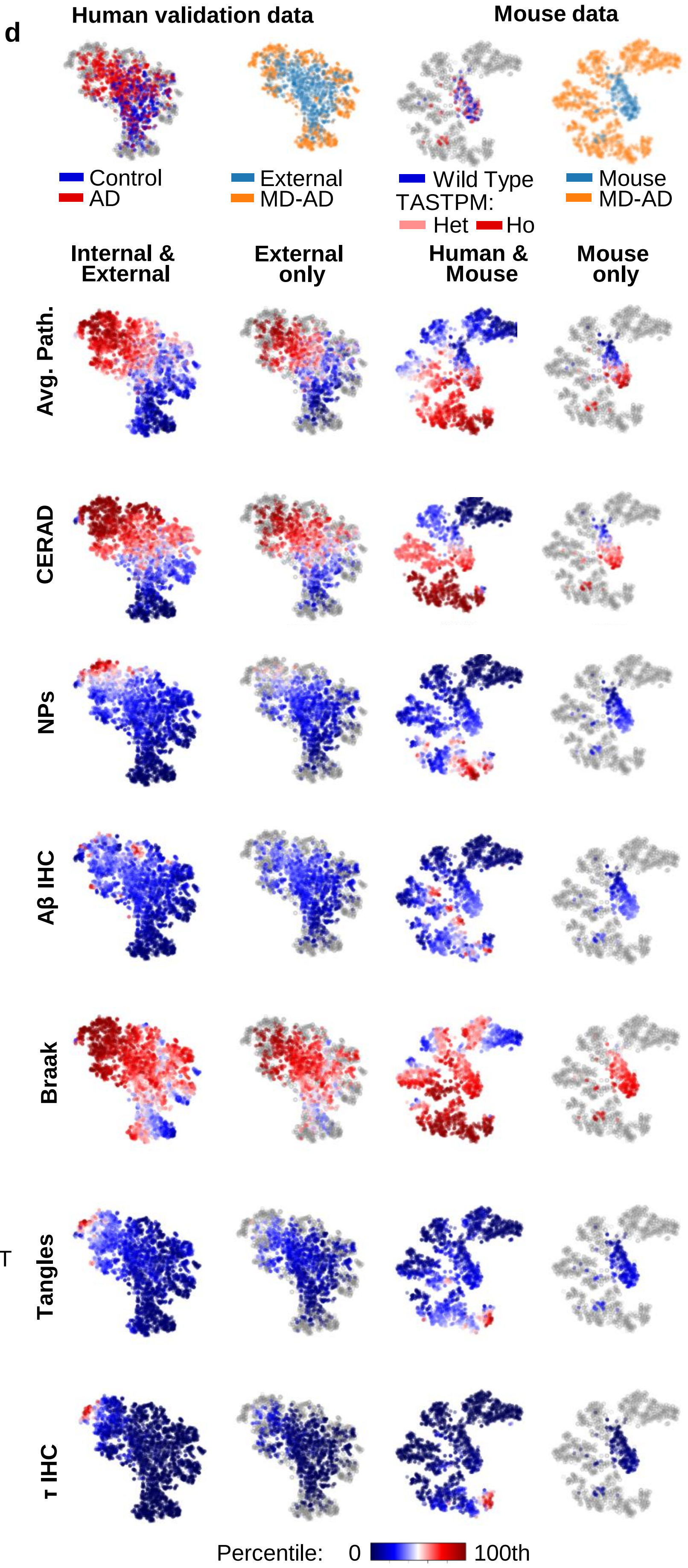

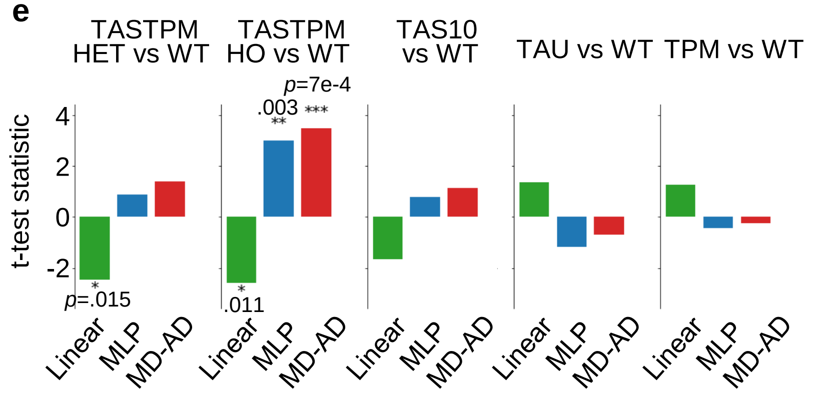

Supplementary Figure 4. (**a**) *t*-test statistics measuring differences between each model's predicted neuropathology scores for AD-diagnosed vs. control individuals. (b) Significance of 2-sided *t*-tests measuring between-group differences as shown in boxplots in Figure 2c. (**c**) Significance of 2-sided *t*-tests measuring between-group differences as shown in boxplots in Figure 2d. (**d**) t-SNE plots of embedded samples for external and mouse data sets. **(e)** *t*-test statistics measuring differences between each model's predicted neuropathology scores for AD model strains of mice vs. wild type (WT) mice.

a

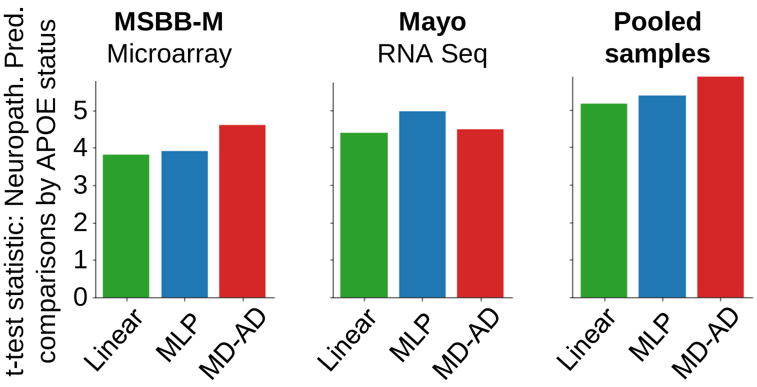

d

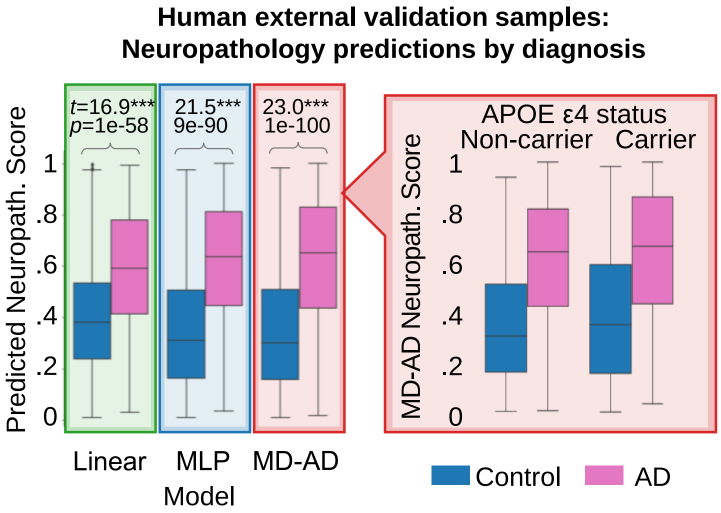

b

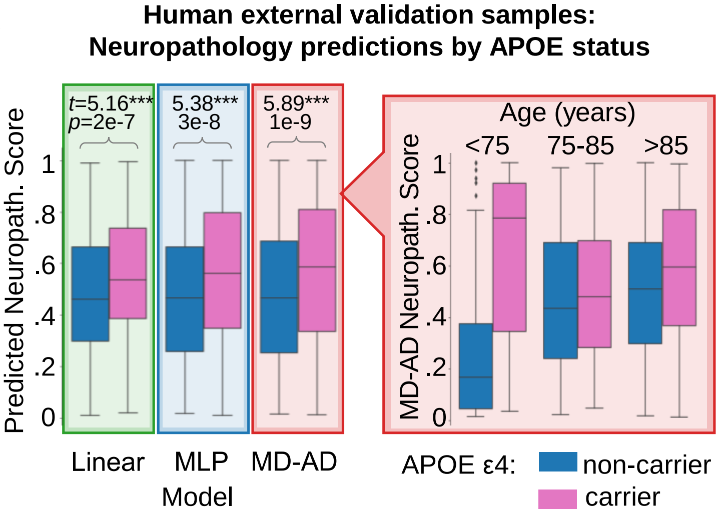

e

		Non-carrier		ε4 carrier	
		C	AD	C	AD
Non-carrier	C (n=406)		2e-48	.019	5e-52
	AD (n=320)	2e-48		3e-17	
ε4 carrier	C (n=144)	.019	3e-17		1e-18
	AD (n=317)	5e-52		1e-18	

t-test p-value: <.05 <.01 <.001 Not significant

c

		<75		75-85		>85	
		NC	ε4	NC	ε4	NC	ε4
<75	Non-carrier (n=71)		1E-12	5E-7	3E-9	1E-13	8E-18
	ε4 carrier (n=72)	1E-12		2E-5	4E-4	7E-6	
75-85	Non-carrier (n=125)	5E-7	2E-5				3E-5
	ε4 carrier (n=143)	3E-9	4E-4				.002
>85	Non-carrier (n=530)	1E-13	7E-6				2E-5
	ε4 carrier (n=246)	8E-18		3E-5	.002	2E-5	

Supplementary Figure 5. External validation results considering APOE status. (a-c) show how neuropathology predictions compare for carriers vs. non-carriers of the APOE ε4 allele. **(a)** t-test statistics measuring differences between each model's predicted neuropathology scores for carriers vs. non-carriers of APOE ε4. **(b)** For samples from external validation data sets, we obtain neuropathology scores for each sample from each model. *Left*: Box plots displaying the distribution of predicted neuropathology scores from each method for APOE ε4 carriers vs. non-carriers. *T*-tests highlight between-group differences for each method (two-sided *t*-test, ****p*<.001; see sample sizes in part c.) *Right*: Box plots displaying the distribution of MD-AD's predicted neuropathology scores split by age group and APOE status. All box plots in this figure indicate median (center line), upper and lower quartiles (box limits), 1.5x interquartile range from quartiles (whiskers), and outliers (points). **(c)** sample sizes and *p*-values from *t*-tests comparing pairs of groups shown in part b.). **(d)** *Left*: Predicted neuropathology scores for each method split by diagnosis (replicated from Figure 2c; see sample sizes in part e.). *Right*: Box plots displaying the distribution of MD-AD's predicted neuropathology scores split by both diagnosis and APOE status (same box plot elements as described in part b). **(e)** sample sizes and *p*-values from two-sided t-tests comparing pairs of groups shown in part d.

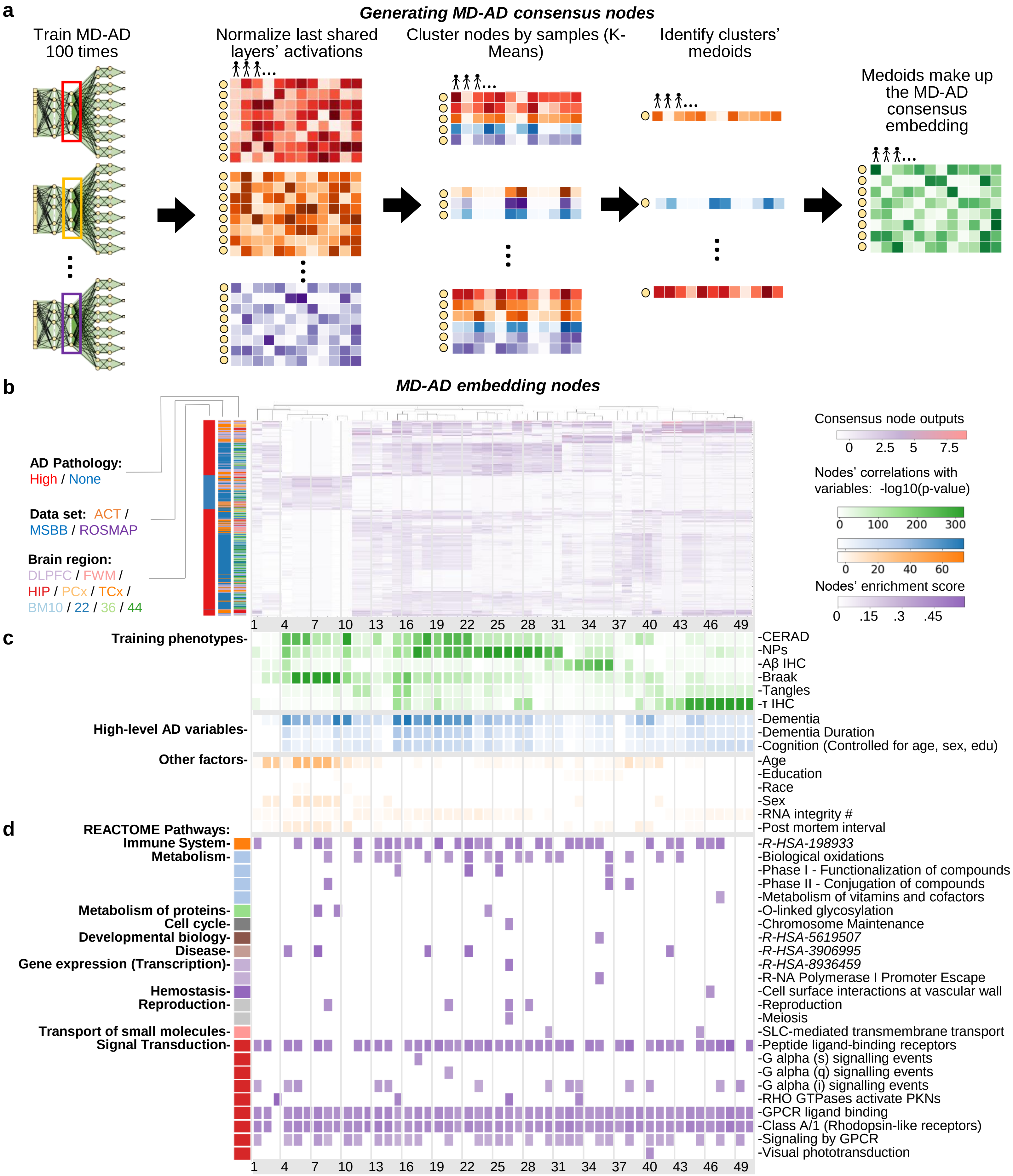

Supplementary Figure 6. Generating and annotating MD-AD "consensus" nodes. **(a)** Illustration of how we generate MD-AD consensus nodes. **(b)** Bi-clustered last shared layer consensus node embeddings. **(c)** Correlations between consensus nodes and phenotypes of interest. Cells show the –log10(*p*-value) of the correlation after FDR correction across nodes. **(d)** Nodes' GSEA enrichment score (ES) for REACTOME pathways: For each node, we obtain integrated gradients weights from each gene. We display only cells with |ES|>.2 and *p*<.05 after FDR correction (across nodes). REACTOME pathways with long names are indicated by their REACTOME stable IDs.

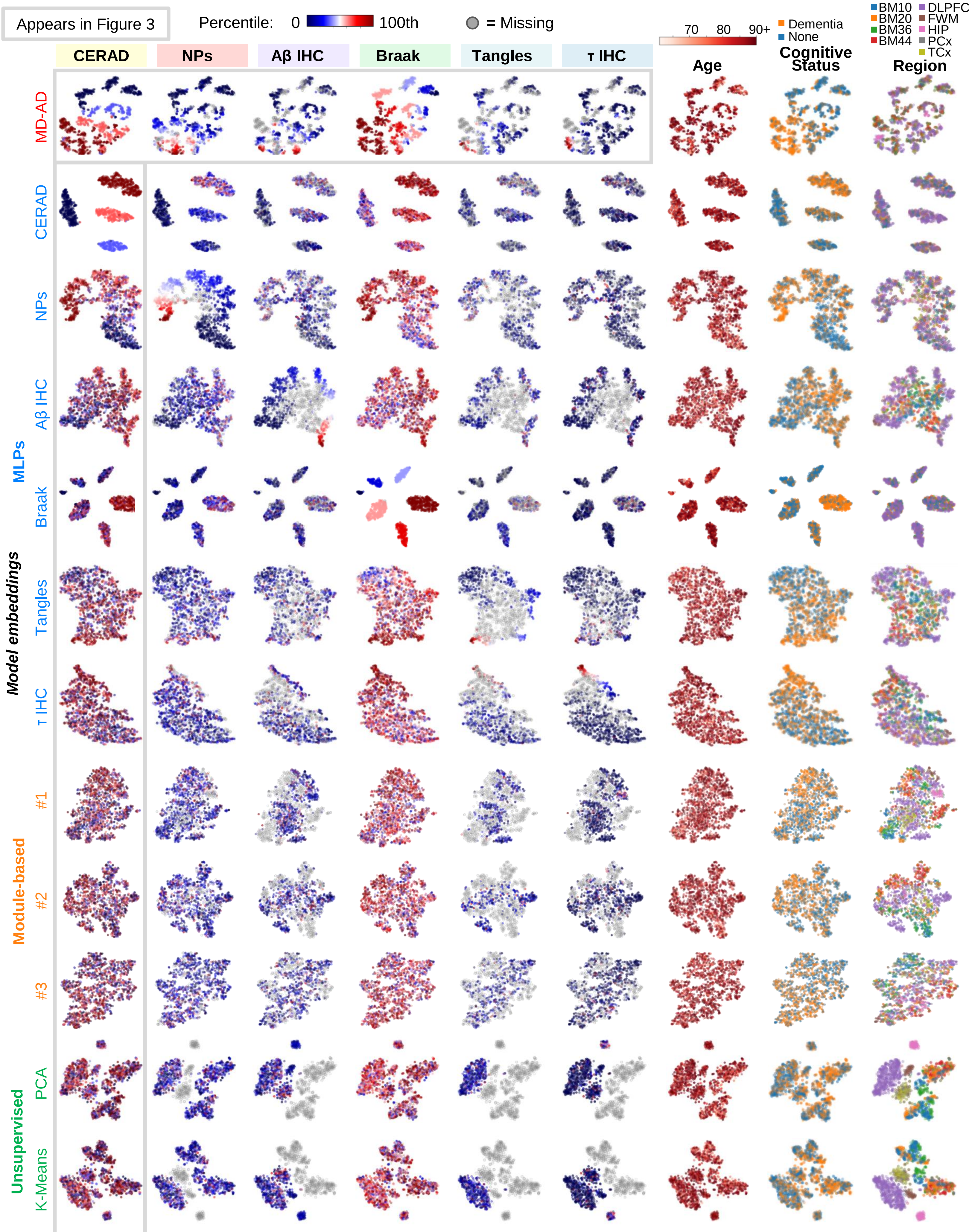

Supplementary Figure 7. *t*-SNE representation of all methods' embeddings colored by pathology values.

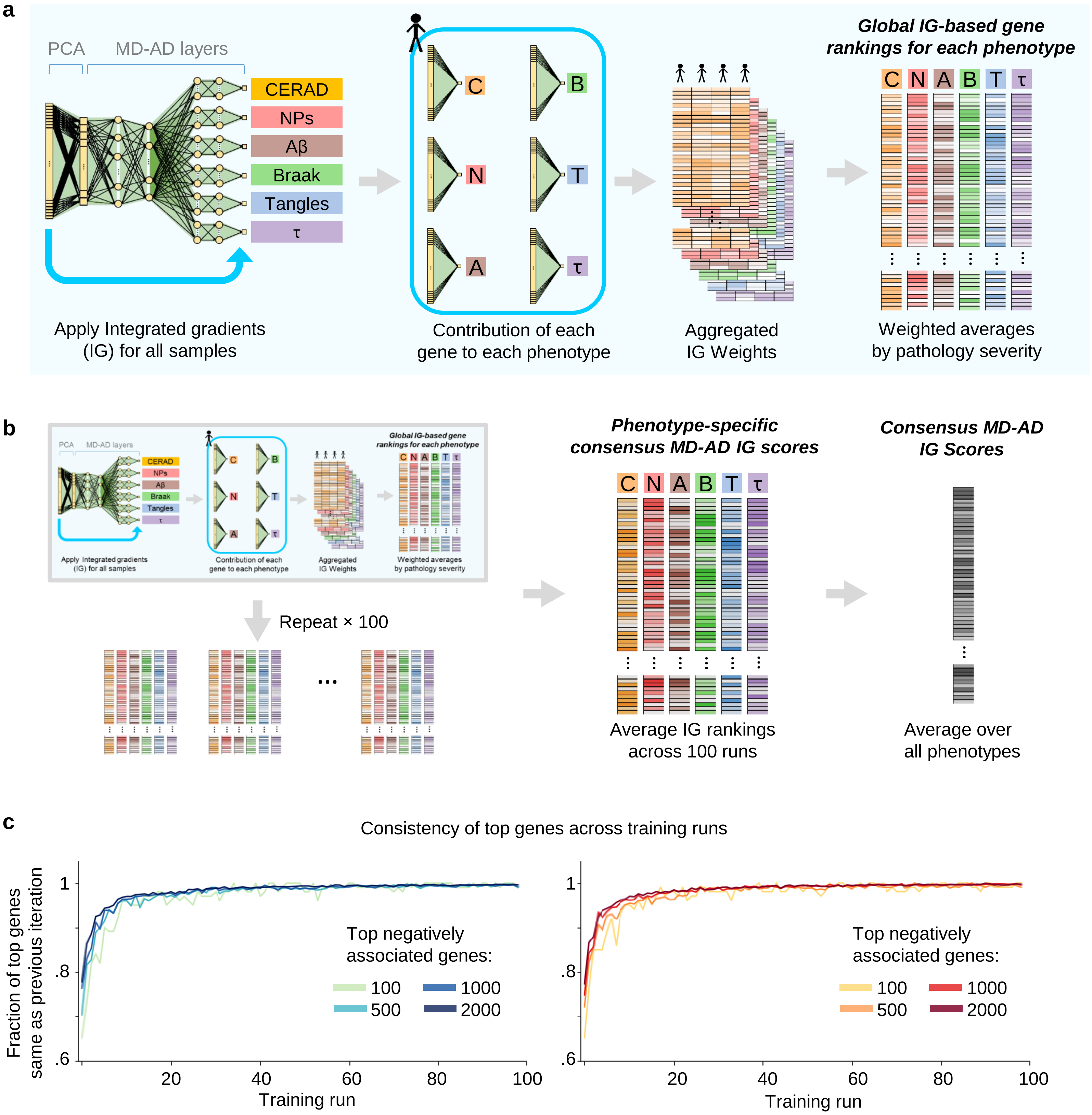

Supplementary Figure 8. Illustration of how MD-AD "consensus" gene scores are generated. **(a)** For a single training run, we aggregate IG scores across samples to obtain a ranking over genes for each phenotype. **(b)** We aggregate IG gene scores across 100 re-trainings of MD-AD. **(c)** Consistency of top genes from aggregating IG gene scores across multiple training runs.

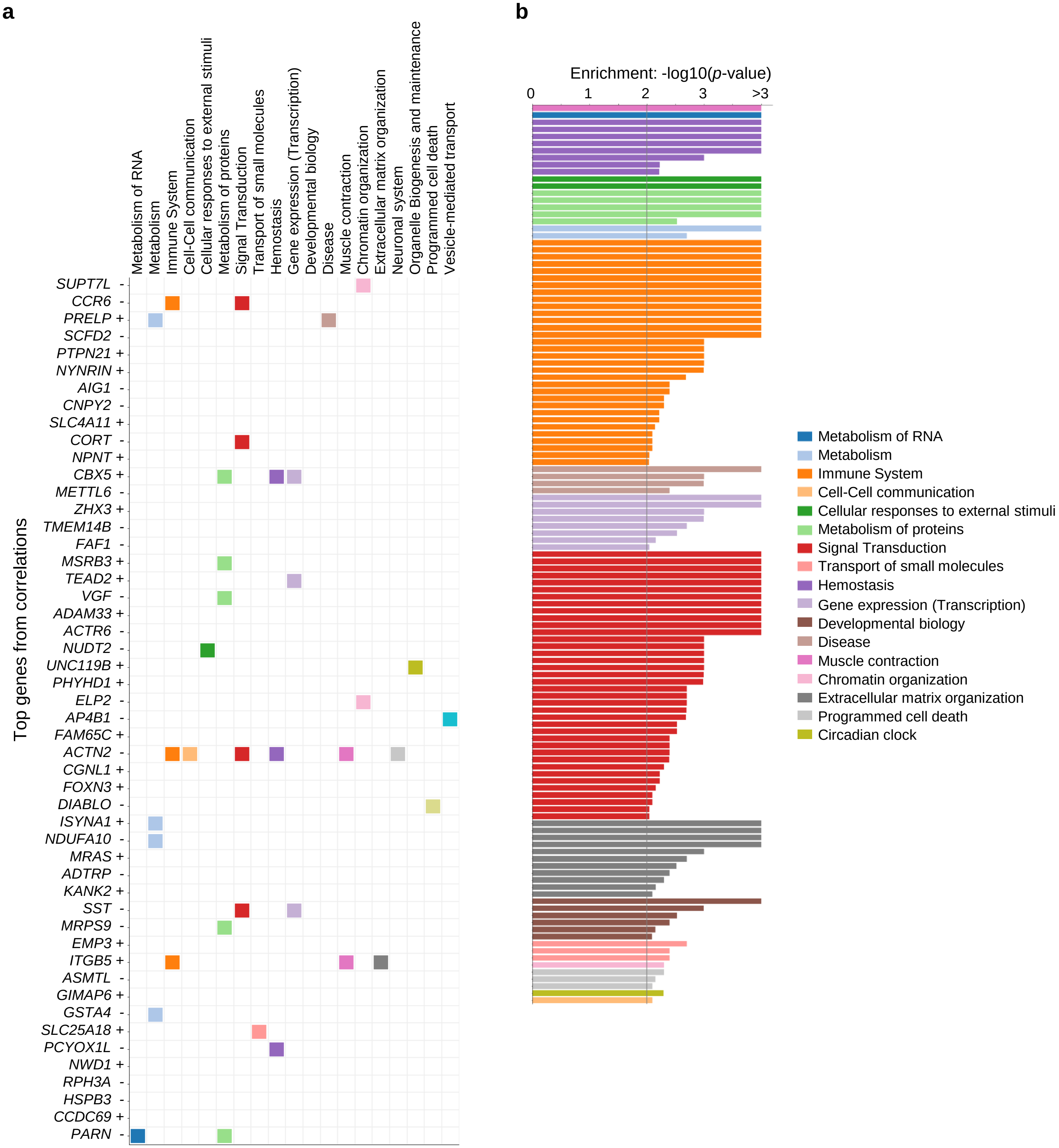

Supplementary Figure 9. Top genes and REACTOME pathway enrichment for correlation-based ranking: (**a**) Top 50 genes ranked by correlations between expression and pathology. (**b**) GSEA enrichment *p*-values for pathways enriched in correlation-based ranking.

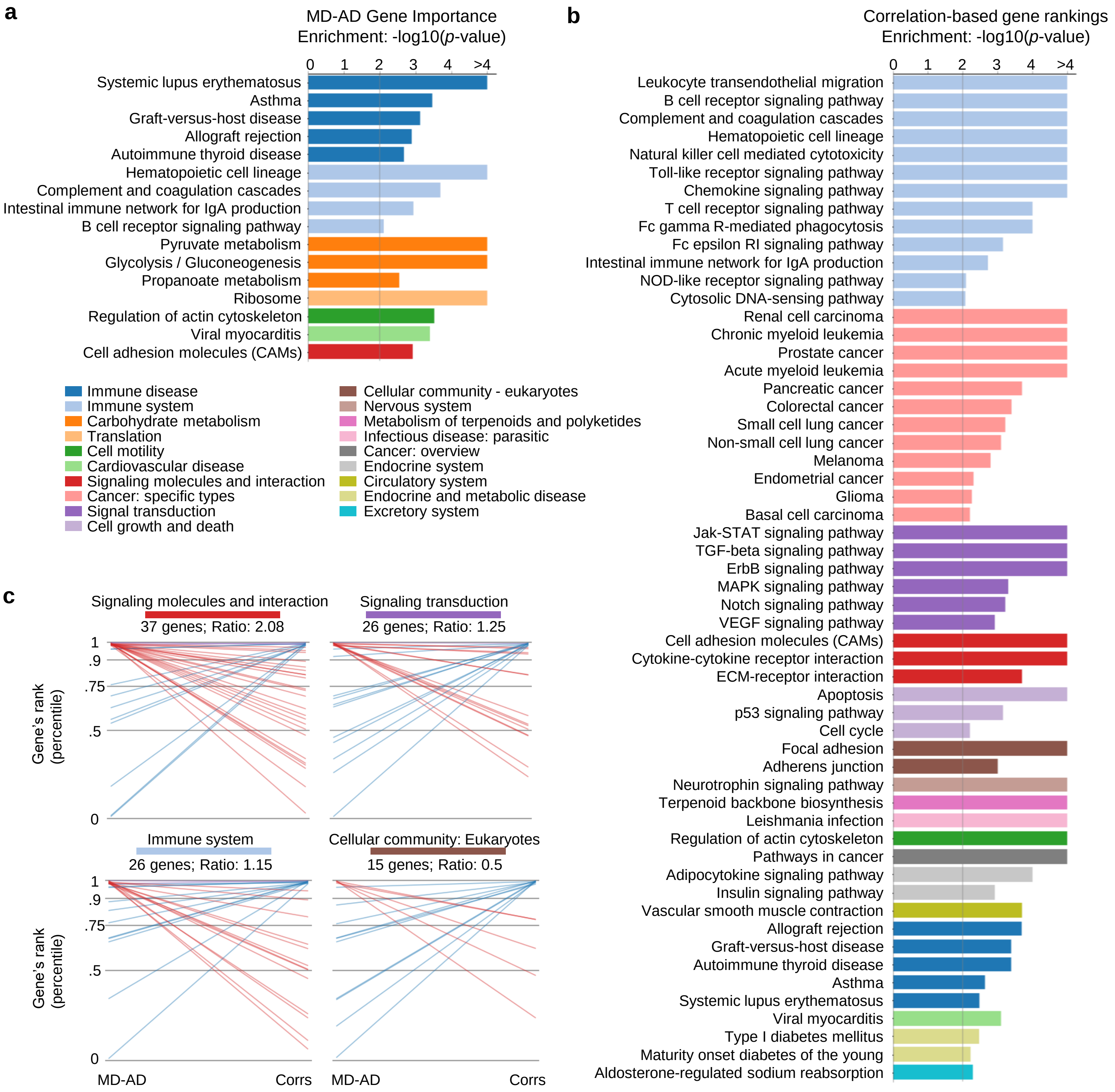

Supplementary Figure 10. (**a**) GSEA enrichment *p*-values for **KEGG** pathways enriched in MD-AD gene ranking, (**b**) GSEA enrichment *p*-values for pathways enriched in correlation-based ranking, (**c**) Comparison of top genes for MD-AD vs correlations. For MD-AD and correlation-based rankings, we identify all genes in the top 2% of the ranking, and then check their membership in KEGG categories.

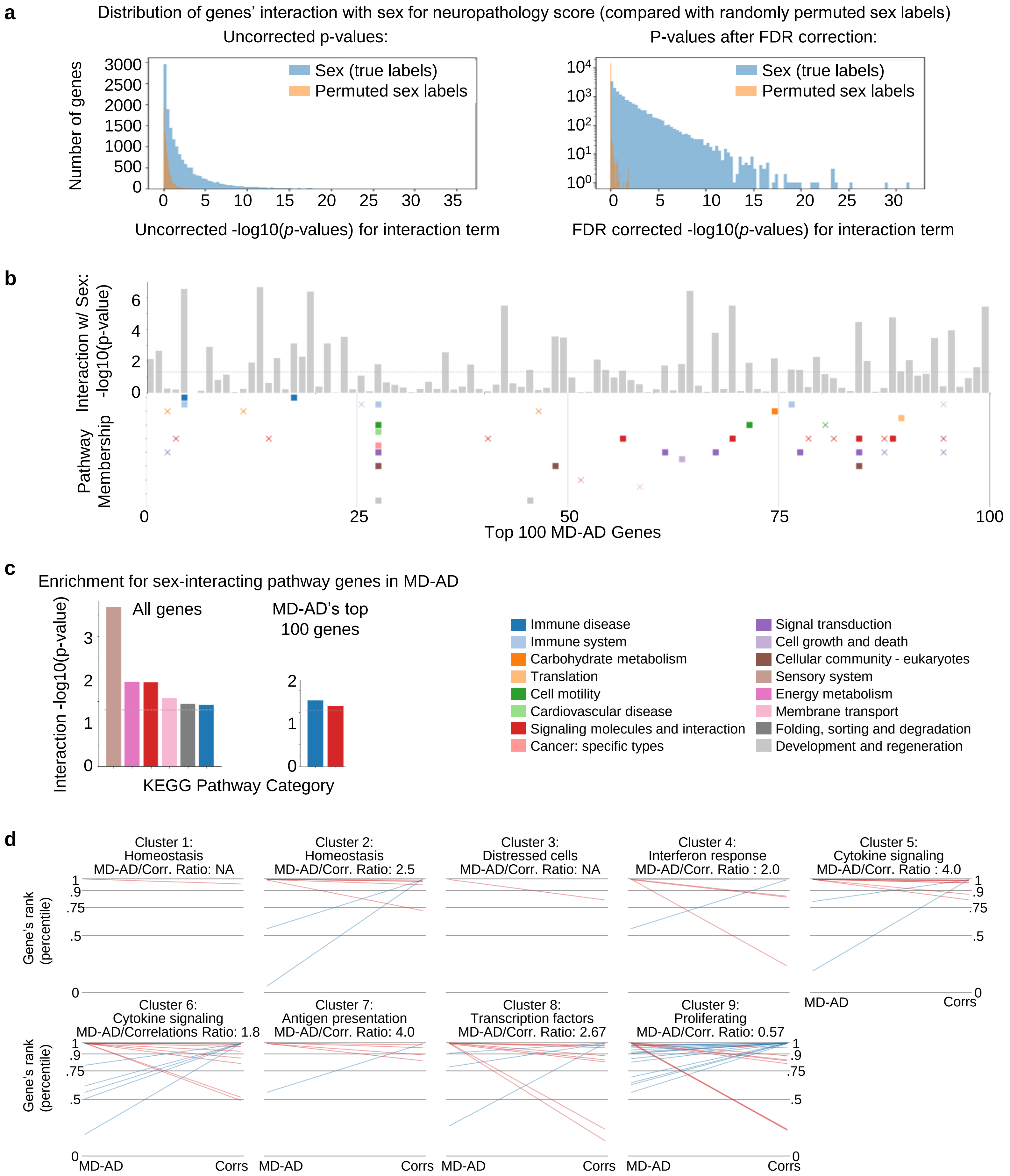

Supplementary Figure 11. Additional details for sex interaction results. **(a)** Distribution of genes' interactions with sex for MD-AD scores. For each gene, we compute the –log10(p-value) for the interaction term between the gene's expression and sex. For comparison, we show the distribution from an experiment with all sex labels shuffled across samples. **(b)** Replicated results from **Figure 5a** with KEGG categories. **(c)** Replicated results from **Figure 5b** with KEGG categories. **(d)** Comparison of top 1% ranked genes from MD-AD vs a correlation-based approach for microglial cluster members.

a

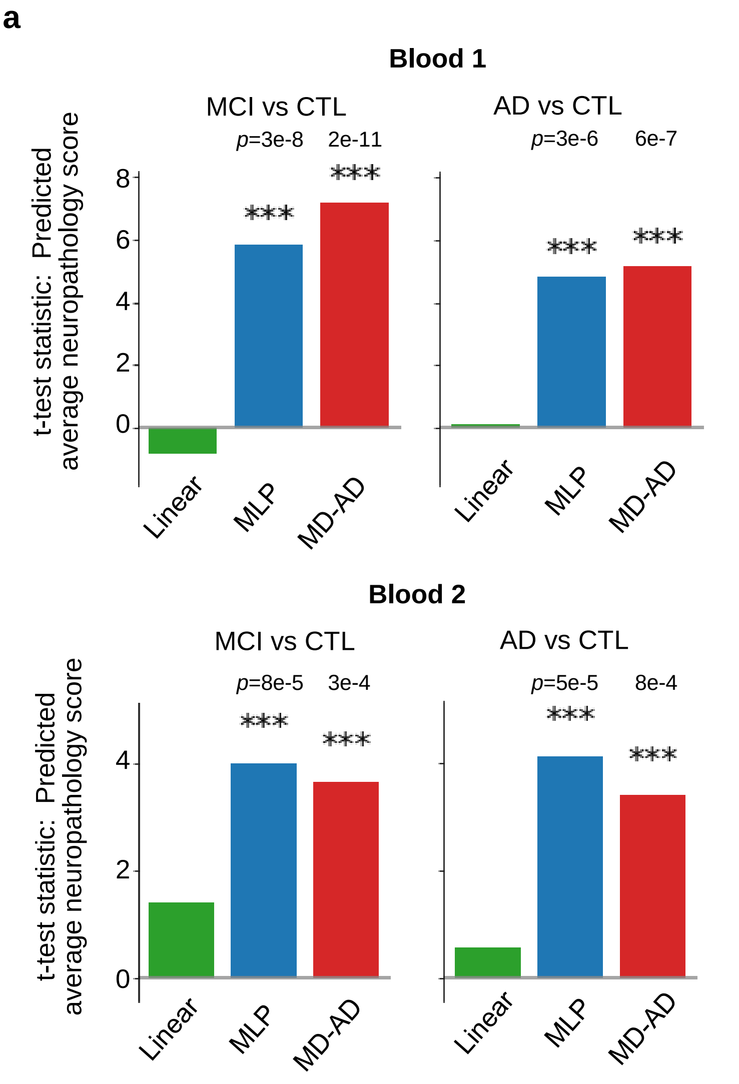

b

t-test p-value: <.05 <.01 <.001 Not significant

		<74			[74-80)			80+		
	Group (N)	CTL	MCI	AD	CTL	MCI	AD	CTL	MCI	AD
<74	CTL (n=113)		1E-8	5E-7		8E-8	7E-5		3E-5	1E-5
	MCI (n=64)	1E-8			7E-6		.042	.003		
	AD (n=89)	5E-7			3E-4			.029		
[74-80)	CTL (n=82)		7E-6	3E-4		3E-5	.01		.003	.004
	MCI (n=63)	8E-8			3E-5			.007		
	AD (n=90)	7E-5	.042		.01					
80+	CTL (n=43)		.003	.029		.007				
	MCI (n=62)	3E-5			.003					
	AD (n=105)	1E-5			.004					

c

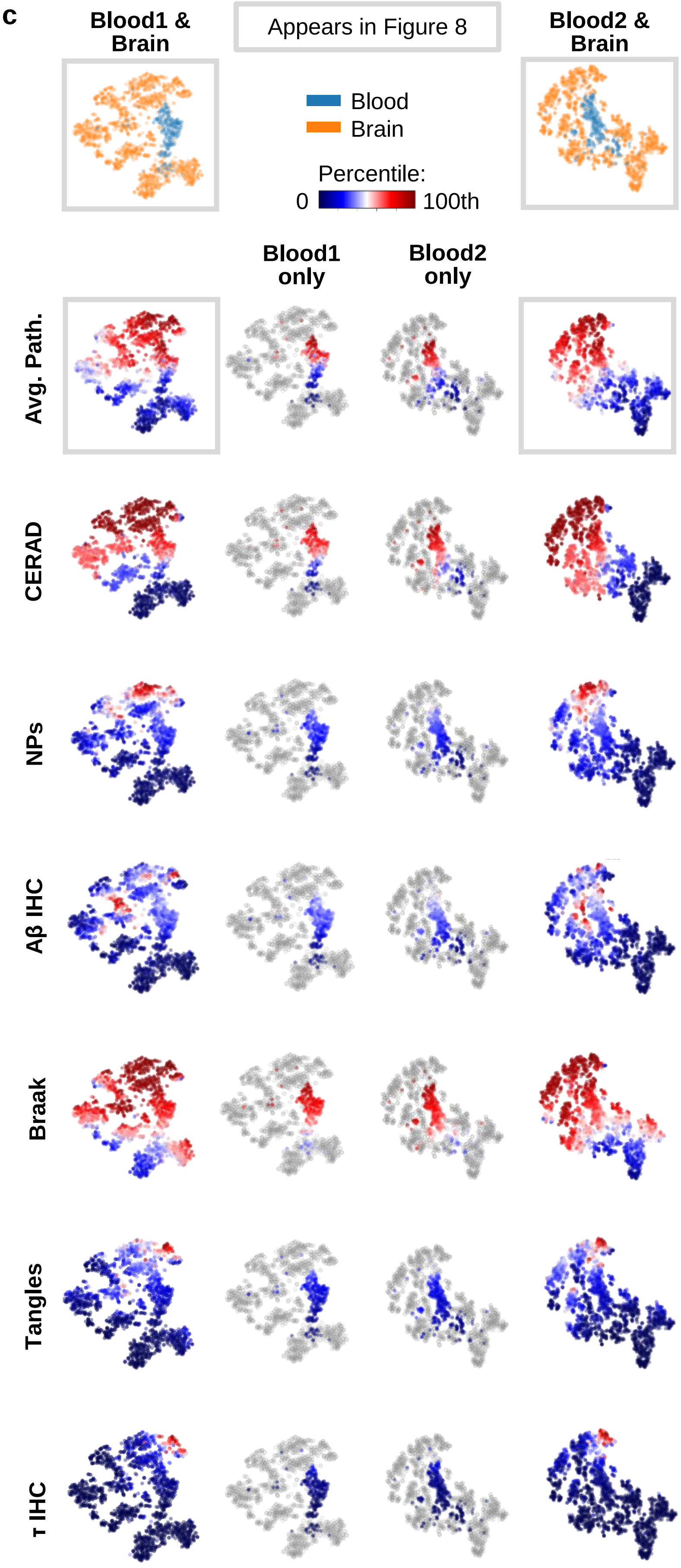

Supplementary Figure 12. (**a**) *T*-test statistics for comparisons among MD-AD predicted neuropathology and cognitive states, separately for blood datasets (see sample sizes in part b). (**b**) Pair-wise *t*-test *p*-values from Figure 7b. **(c)** Embeddings from MD-AD's last shared layer for brain and blood data. Each plot is colored by dataset or predicted pathology severity for various phenotypes. Pathology severity plots are produced with and without MD-AD training (brain) samples for clarity.

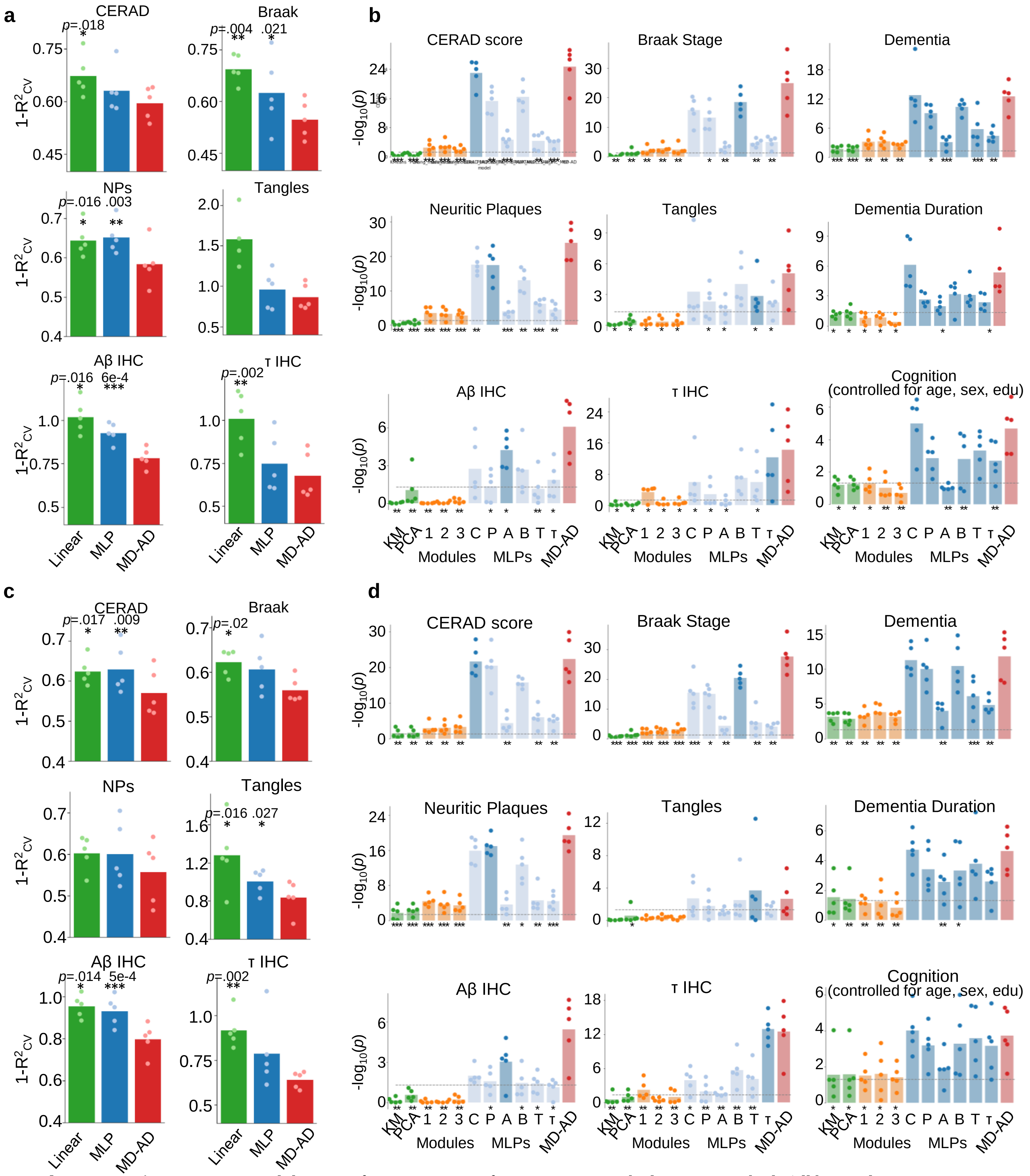

Supplementary Figure 13. Cross-validation performance metrics for experiments with alternative methods (all bars indicate an average over five test runs, and each run's performance is overlaid as a dot). For each subplot, we highlight differences in performance between alternative methods and MD-AD via 2-sided paired *t*-tests using n=5 test runs (*p*-values: *<.05, **<.01, ***<.001). **(a)** Figure 2a replicated with results from training and evaluation using GE normalized to account for post-mortem interval (PMI) and RNA integrity number (RIN). **(b)** Figure 3a replicated with results from training and evaluation using GE normalized to account for PMI and RIN. Each bar represents the –log10(p-value) after FDR correction over nodes for the correlation between the best node and phenotype listed, averaged over 5 test run. **(c)** Figure 2a replicated with new cross-validation and test splits generated by pseudorandomly assigning samples by individual (rather than completely randomly). **(d)** Figure 3a replicated with the new cross-validation and test splits.
