# Supplementary material for: Unified AI framework to uncover deep interrelationships between gene expression and Alzheimer’s disease neuropathologies
Source: Nat Commun. 2021 Sep 10;12:5369. doi: 10.1038/s41467-021-25680-7 (PMC8433314; doi:10.1038/s41467-021-25680-7)
Supplement: Supplementary file 8 — Description of Additional Supplementary Files [file 41467_2021_25680_MOESM8_ESM.pdf]

Description of Additional Supplementary Files

File Name: Supplementary Data 1
Description: Descriptions of the six neuropathological phenotypes used for training MD-AD (and baseline models). We provide descriptions for each phenotype directly from documentation from each individual cohort study.

File Name: Supplementary Data 2
Description: Overview of cohorts used for MD-AD training. For continuous variables, we show mean and standard deviation and make pairwise comparisons using two-sided *t*-tests. For categorical or binary variables, show percentages and use chi-square tests to compare across cohorts. For MD-AD training phenotypes, we show the mean and standard deviation (along with ranges due to skewed distributions) of the values consistent with units as indicated in Supplementary Data 1 (note that units vary across studies). Because units varied across studies, for computing statistical comparisons between MD-AD training phenotypes, we consider the normalized values used in MD-AD model training (values normalized to range from 0 to 1 within each data set). "NA" indicates that the variable was not available for the dataset. Across all variables, significant differences between groups (calculated via statistical tests described above) are indicated by the first letter of the dataset (e.g., $^{A}p<.05$, $^{AA}p<.01$, $^{AAA}p<.001$).

*For MSBB, cognition status was based on the clinical dementia rating (CDR). For ROSMAP, this was based on consensus between a neuropsychologist and clinician based on a battery of tests.

**For NINCDS/ADRDA criteria, Possible and Probable AD both indicates that the individual has dementia, but with differing certainty about the cause while they were alive.

File Name: Supplementary Data 3
Description: Overview of MD-AD consensus nodes. Each node is annotated with -log10(p-values) for phenotypes and covariates of interest. We also show or enrichment scores for KEGG and REACTOME pathways. Enrichment scores were masked to 0 when the enrichment p-value were not significant (i.e., $p>0.05$ after Bonferroni correction across nodes).

File Name: Supplementary Data 4
Description: MD-AD's consensus gene scores, with high scores being most positively related to neuropathology and low scores being most negatively related to neuropathology. "all-related" is averaged over the six neuropathological phenotypes, while "abeta-related" is averaged over CERAD, PLAQUES, and ABETA_IHC, and "tau-related" is averaged over BRAAK, TANGLES, and TAU_IHC.

File Name: Supplementary Data 5
Description: Overview of AddNeuroMed blood samples. For each cell, we show summary statistics (and

any group with which this group has a statistically significant difference, as measured by independent t-tests or chi-square tests). For each group, we highlight all groups that are significantly different (*p<.05, **p<.01, ***p<.001).

File Name: Supplementary Data 6

Description: Left: Final selected models for MD-AD and baseline methods, after aggregating performance across all 5 test folds. Right: best hyperparameter for each phenotype within each model class, separately computed for each training/test split. All networks were trained for 200 epochs with batch sizes of 20, ReLU activations, and dropout units with 0.1 dropout rate, using an Adam optimizer.
