# Supplementary material for: Unified AI framework to uncover deep interrelationships between gene expression and Alzheimer’s disease neuropathologies
Source: Nat Commun. 2021 Sep 10;12:5369. doi: 10.1038/s41467-021-25680-7 (PMC8433314; doi:10.1038/s41467-021-25680-7)
Supplement: Supplementary file 10 — Reporting Summary [file 41467_2021_25680_MOESM10_ESM.pdf]

nature research

Corresponding author(s): Su-In Lee & Sara Mostafavi

Last updated by author(s): Jan 4, 2021

Reporting Summary

Nature Research wishes to improve the reproducibility of the work that we publish. This form provides structure for consistency and transparency in reporting. For further information on Nature Research policies, see our Editorial Policies and the Editorial Policy Checklist.

Statistics

For all statistical analyses, confirm that the following items are present in the figure legend, table legend, main text, or Methods section.

n/a	Confirmed	
[ ]	[x]	The exact sample size (*n*) for each experimental group/condition, given as a discrete number and unit of measurement
[x]	[ ]	A statement on whether measurements were taken from distinct samples or whether the same sample was measured repeatedly
[ ]	[x]	The statistical test(s) used AND whether they are one- or two-sided<br>*Only common tests should be described solely by name; describe more complex techniques in the Methods section.*
[ ]	[x]	A description of all covariates tested
[ ]	[x]	A description of any assumptions or corrections, such as tests of normality and adjustment for multiple comparisons
[ ]	[x]	A full description of the statistical parameters including central tendency (e.g. means) or other basic estimates (e.g. regression coefficient) AND variation (e.g. standard deviation) or associated estimates of uncertainty (e.g. confidence intervals)
[ ]	[x]	For null hypothesis testing, the test statistic (e.g. *F*, *t*, *r*) with confidence intervals, effect sizes, degrees of freedom and *P* value noted<br>*Give P values as exact values whenever suitable.*
[x]	[ ]	For Bayesian analysis, information on the choice of priors and Markov chain Monte Carlo settings
[ ]	[x]	For hierarchical and complex designs, identification of the appropriate level for tests and full reporting of outcomes
[x]	[ ]	Estimates of effect sizes (e.g. Cohen's *d*, Pearson's *r*), indicating how they were calculated

Our web collection on statistics for biologists contains articles on many of the points above.

Software and code

Policy information about availability of computer code

Data collection: No software was used.

Data analysis: All code is available at https://github.com/suinleelab/MD-AD.

Our code relies on the following standard Python software packages: numpy (1.17.3), pandas (0.24.1), scipy (1.3.1), matplotlib (3.1.2), seaborn (0.9.0), h5py (2.9.0)
We additionally used the following Python software packages:
- Tensorflow (1.3.0), Keras (2.0.4) were used for constructing deep learning models.
- Scikit-learn (0.21.3) was used for data dimensionality reduction (e.g., principal components analysis, t-SNE algorithm).
- IntegratedGradients (used for feature attribution calculations) - https://github.com/hiranumn/IntegratedGradients
- GSEApy (used for gene set enrichment analyses) - https://pypi.org/project/gseapy/
- ComBat (used for batch effect correction) - https://github.com/brentp/combat.py

For manuscripts utilizing custom algorithms or software that are central to the research but not yet described in published literature, software must be made available to editors and reviewers. We strongly encourage code deposition in a community repository (e.g. GitHub). See the Nature Research guidelines for submitting code & software for further information.

Data

Policy information about availability of data

All manuscripts must include a data availability statement. This statement should provide the following information, where applicable:

- Accession codes, unique identifiers, or web links for publicly available datasets
- A list of figures that have associated raw data
- A description of any restrictions on data availability

No new data are generated in this study. All data sets used were either publically available or available subject to data-use terms and conditions as described below. Most human brain gene expression and phenotype data sets were obtained via the AD Knowledge Portal Synapse platform (doi: 10.7303/syn2580853). Access to these data sets may only be obtained after registering for a Synapse.org account, agreeing to acknowledge data used in any publications, and submitting a data use certificate (separately as needed for each data set). Our study uses the following data sets (with listed Synapse IDs; URLs): ACT (syn5759376; https://adknowledgeportal.synapse.org/Explore/Studies/DetailsPage?Study=syn5759376), ROSMAP (syn3219045; https://adknowledgeportal.synapse.org/Explore/Studies/DetailsPage?Study=syn3219045; doi: 10.1038/s41593-018-0154-9), MSBB (RNA Sequencing: syn3159438; https://adknowledgeportal.synapse.org/Explore/Studies/DetailsPage?Study=syn3159438, Microarray: syn3157699; https://adknowledgeportal.synapse.org/Explore/Studies/DetailsPage?Study=syn3157699), Mayo Clinic Brain Bank (syn5550404; https://adknowledgeportal.synapse.org/Explore/Studies/DetailsPage?Study=syn5550404; doi: 10.1038/sdata.2016.89).

All other human brain, mouse brain, and human blood data sets were downloaded from the Gene Expression Omnibus (GEO). The following data sets are publically available for download (with listed accession codes; URLs): HBTRC (GSE44772; https://www.ncbi.nlm.nih.gov/geo/query/acc.cgi?acc=GSE44772; doi: 10.1016/j.cell.2013.03.030), human blood gene expression and phenotype data from the AddNeuroMed cohort (GSE63060; https://www.ncbi.nlm.nih.gov/geo/query/acc.cgi?acc=GSE63060 and GSE63061; https://www.ncbi.nlm.nih.gov/geo/query/acc.cgi?acc=GSE63061), Mouse brain gene expression samples and associated phenotypes (GSE64398; https://www.ncbi.nlm.nih.gov/geo/query/acc.cgi?acc=GSE64398).

Our study reports pathway enrichment for our results with respect to publically available gene sets. These include REACTOME and KEGG pathways available from MSigDB (c2 pathways v7.0; http://www.gsea-msigdb.org/gsea/msigdb/genesets.jsp?collection=C2). We also compared our results with gene signatures from Olah et al.38 (Supplementary Data 5 in their publication) and Mathys et al.8 (Supplementary Table 6 in their publication).

Source data for replicating all figures are provided with this paper.

Field-specific reporting

Please select the one below that is the best fit for your research. If you are not sure, read the appropriate sections before making your selection.

☒ Life sciences ☐ Behavioural & social sciences ☐ Ecological, evolutionary & environmental sciences

For a reference copy of the document with all sections, see nature.com/documents/nr-reporting-summary-flat.pdf

Life sciences study design

All studies must disclose on these points even when the disclosure is negative.

Sample size	For human brain gene expression data, n=3,300. We used all samples for which gene expression and phenotype data are available. These samples represent all subjects with available frozen brain samples at the time of data generation. Of the 3,300 total samples, 1,758 were used for the development of the MD-AD model (ACT, ROSMAP, and MSBB RNA-Seq data sets). The remaining 1,542 samples were used for external validation (Mayo clinic brain bank RNA-Seq data and HBTRC and MSBB microarray data sets). Finally, to provide further external validation for our method, we sought an additional animal model dataset, as well as an additional dataset from another tissue. These would indicate whether it is possible for MD-AD to transfer across species or tissues. Thus, we used 138 mouse brain gene expression samples from Matarin et al., 2015, and 711 human blood samples from the AddNeuroMed cohort.
Data exclusions	All data meeting pre-determined quality control criteria were included for analysis. For brain gene expression data, only cortical samples were used.
Replication	MD-AD model performance was externally validated using 1,542 separate brain gene expression samples, 711 human blood samples, and 138 mouse brain gene xpression samples.
Randomization	All human data was obtained via observational cohort studies, thus, randomization does not apply. Covariates were not controlled as a pre-processing step and the model was allowed to learn any implicit covariates contained in gene expression data. Instead, we use post-hoc analyses to identify covariate effects in the model. For mouse studies, data were obtained from transgenic lines along with littermate controls to minimize the presence of covariate effects (furthermore, no model training was based on mouse data - it was only used for evaluation).
Blinding	Samples were collected and measures by investigators blinded to groups/phenotypes for all brain, blood, and mouse datasets. Preprocessing of datasets was blinded to group labels. Models were trained to predict phenotypes and thus group labels were used for training; however, extensive internal and external validation was performed.

Reporting for specific materials, systems and methods

We require information from authors about some types of materials, experimental systems and methods used in many studies. Here, indicate whether each material, system or method listed is relevant to your study. If you are not sure if a list item applies to your research, read the appropriate section before selecting a response.

Materials & experimental systems

n/a	Involved in the study
☒	☐ Antibodies
☒	☐ Eukaryotic cell lines
☒	☐ Palaeontology and archaeology
☒	☐ Animals and other organisms
☒	☐ Human research participants
☒	☐ Clinical data
☒	☐ Dual use research of concern

Methods

n/a	Involved in the study
☒	☐ ChIP-seq
☒	☐ Flow cytometry
☒	☐ MRI-based neuroimaging
