# Supplementary material for: Unified AI framework to uncover deep interrelationships between gene expression and Alzheimer’s disease neuropathologies
Source: Nat Commun. 2021 Sep 10;12:5369. doi: 10.1038/s41467-021-25680-7 (PMC8433314; doi:10.1038/s41467-021-25680-7)
Supplement: Supplementary file 11 — Source Data [file 41467_2021_25680_MOESM11_ESM.zip › sourcedata/README.docx]

Below, we provide details about each of the csv files provided as source data for our display items. We have broken down our explanations by subfigure, and indicate which csv files correspond to them (along with details about each column in the csv file).

Figure 2a

**Files: *2a_cv_test_performance.csv***

**Columns:**

- 1-R^2_CV for predicted phenotypes: CERAD, BRAAK, PLAQUES, TANGLES, ABETA_IHC, TAU_IHC
- Model identifiers: Model type, Fold

Figure 2b

**Files: *2b_cv_rosmap_subsets.csv***

**Columns:**

- Val – 1-R^2_CV for predicted phenotype using only ROSMAP test samples
- Phenotype – Phenotype predicted in this row
- Fold - Which of the five test splits was used for training/evaluation
- Subset – Which of the three datasets whose training samples were included during training (A=ACT, M=MSBB, R=ROSMAP)

Figures 2c, 2d, 7a-b

**Files: *2c_ext_val_human_brain.csv, 2d_ext_val_mouse.csv, 7ab_ext_val_human_blood.csv***

**Columns:**

Each file has specific characteristics relevant to each population:

- ***2c_ext_val_human_brain.csv***: AD (postmortem AD diagnosis), age_groups
- ***2d_ext_val_mouse.csv***: strain, age, region
- ***7ab_ext_val_human_blood.csv***: cognitive status (CTL=cognitively normal, MCI=mild cognitive impairment, AD=dementia), age_groups

The last three columns for these files contain the predicted neuropathology scores generated from each of the three methods (linear baseline, MLP baselines, MD-AD): Linear_predicted_neuropath_score, MLP_baselines_predicted_neuropath_score, MD-AD_predicted_neuropath_score

Figure 3a-b (bar charts):

**Files: *3ab_embedding_correlations.csv***

**Columns:**

- model (models evaluated),
- test_set (which of five test sets is being evaluated, after training on the corresponding training set)
- Correlation p-value of the best node with each of the following phenotypes: CERAD, BRAAK, PLAQUES, TANGLES, ABETA_IHC, TAU_IHC, addx_to_death_cats (high level phenotype – bins for time between dementia diagnosis and death), controlled_cogn_global_last (most recent cognition score controlling for age, sex, and education), dementia (binary label – 1 if they have diagnosed dementia, 0 otherwise)

Figure 3a, 3c (t-SNE plots)

**Files: *3ac_internal_tsne_embeddings.csv***

**Columns:**

Columns are E1_...and E2_..., which represent the first two t-SNE embedding dimensions from embeddings generated by each of the methods displayed in Figure 3:

- MD-AD: MDAD-consensus
- MLPs: MLP-CERAD, MLP-BRAAK, MLP-PLAQUES, MLP-TANGLES, MLP-ABETA_IHC, MLP-TAU_IHC
- Modules from prior work: modules-zhang_et_al_2013, modules-mostafavi_et_al_2018, modules-logsdon_et_al_2019
- Unsupervised embedding approaches: PCA-50, KMeans-50

Figures 3d, 3e, 7c

**Files: *3d_external_tsne_embeddings_human_brain.csv, 3e_external_tsne_embeddings_mouse.csv, 7c_external_tsne_embeddings_Blood_GSE63060.csv, 7c_external_tsne_embeddings_Blood_GSE63061.csv***

**Columns:**

- First two columns are always E1 and E2, representing the first two t-_SNE embedding dimensions computed from MD-AD’s last shared layer embedding.
- dataset – “train” if the sample is from the original MD-AD training samples (from ACT, MSBB, ROSMAP), “test” if the sample is from the external validation dataset
- Predicted outputs from the MD-AD model: CERAD_predicted, BRAAK_predicted, PLAQUES_predicted, TANGLES_predicted, ABETA_IHC_predicted, TAU_IHC_predicted
- Averaged phenotype predictions across phenotypes: AB_RELATED_predicted (averaged over CERAD, PLAQUES, ABETA_ICH), TAU_RELATED_predicted (averaged over (BRAAK, TANGLES, TAU_IHC), AVG_predicted (averaged over all 6 phenotypes),
- test_label – label associated with the external validation sample
  - Human brain samples: Control vs AD
  - Mouse samples: strain and age (formatted as strain_agegroup)
  - Blood samples: cognitive status

Figure 4a+c, 6a

**Files: *4ac_gene_ranks_REACTOME.csv, 6a_gene_ranks_microglial_clusters.csv***

**Columns:**

- gene (in gene symbol format)
- ranked_genes_MD-AD and ranked_genes_correlations – percentile gene rankings from each method (1=most important, 0=least important)
- gene_sign_MD-AD, gene_sign_correlations – direction of relationship between gene’s expression and neuropathology (“+” indicates that higher expression of the gene is associated with more neuropathology, and “-“ indicates the opposite)
- Remaining columns indicate whether the gene is in the gene set listed as the column name (1 if the gene is in the gene set, 0 otherwise). For ***4ac_gene_ranks_REACTOME.csv***, each column is a REACTOME high-level category. For ***6a_gene_ranks_microglial_clusters.csv***, each column (1-9) is a microglial cluster described in Olah et al., 2020***.***

Figure 4b, 6c (+ Supplementary figures 7b, 8a, 8b)

**Files: *4b_MDAD_finalgenes_enrichment_REACTOME.csv,
6c_MDAD_finalgenes_enrichment_celltypes.csv,*** *supp7b_corr_finalgenes_enrichment_REACTOME.csv, supp8a_MDAD_finalgenes_enrichment_KEGG.csv, supp8b_corr_finalgenes_enrichment_KEGG.csv,*

**Columns:**

- term (gene set) and category (high level category associated with the gene set)
- Enrichment results from GSEA: es (enrichment score), nes (normalized enrichment score), pval (enrichment p-value), fdr (FDR-corrected enrichment p-value), geneset_size, matched_size (gene set size after removing genes not in our dataset)

Figure 5a/5c/6a

**Files: *5ac_6a_IG_interactions_sex_m.csv***

**Columns:**

- gene_symbol
- For each gene, we model the IG score as a linear regression over the gene’s expression level, sample donor’s sex, and the interaction of the two variables. We have columns for the interaction term’s coefficient and p-value (with and without FDR correction over all genes): interaction_coefficient, interaction_pval, interaction_pval_fdr_corrected

Figure 5b, 6b

**Files: *5b_sex_interacting_genes_enrichment_REACTOME_categories.csv, 6b_sex_interacting_genes_enrichment_microglial_genesets.csv***

**Columns:**

We consider two sets of sex-interacting genes: all genes with a significant sex interaction term (p<.05 after FDR correction), and the subset of those genes that are also in the top-100 most important genes. Among both groups, we evaluate the enrichment of each gene set using Fisher’s exact test. We therefore have these columns:

- geneset - either a REACTOME high-level category or microglial cluster (labeled 1-9) described in Olah et al., 2020
- top_100_enrichment_pval – enrichment p-value for the gene set among the sex interacting genes among the top 100 MD-AD genes
- all_genes_enrichment_pval - enrichment p-value for the gene set among all sex interacting genes

Figure 5d

**Files: *5d_gene_vs_IG_KNSTRN.csv, 5d_gene_vs_IG_C4B.csv, 5d_gene_vs_IG_CMTM4.csv, 5d_gene_vs_IG_TREM2.csv, 5d_gene_vs_IG_P2RY11.csv, 5d_gene_vs_IG_SERPINA3.csv***

Each row represents a data sample used to train MD-AD. For each file, we have the following columns relating to the gene indicated in the filename:

- sex_m – sex of the sample’s donor (1=male, 0=female)
- gene_expression – the sample’s expression level for the gene
- gene_IG_score – per-sample IG score for the gene
